# Xenin and Related Peptides: Potential Therapeutic Role in Diabetes and Related Metabolic Disorders

**DOI:** 10.1177/11795514211043868

**Published:** 2021-09-22

**Authors:** Sarah L Craig, Nigel Irwin, Victor A Gault

**Affiliations:** Faculty of Life and Health Sciences, School of Biomedical Sciences, Ulster University, UK

**Keywords:** Xenin-25, glucose-dependent insulinotropic polypeptide, insulin secretion, satiety, hybrid peptides, diabetes

## Abstract

Xenin bioactivity and its role in normal physiology has been investigated by several research groups since its discovery in 1992. The 25 amino acid peptide hormone is secreted from the same enteroendocrine K-cells as the incretin hormone glucose-dependent insulinotropic polypeptide (GIP), with early studies highlighting the biological significance of xenin in the gastrointestinal tract, along with effects on satiety. Recently there has been more focus directed towards the role of xenin in insulin secretion and potential for diabetes therapies, especially through its ability to potentiate the insulinotropic actions of GIP as well as utilisation in dual/triple acting gut hormone therapeutic approaches. Currently, there is a lack of clinically approved therapies aimed at restoring GIP bioactivity in type 2 diabetes mellitus, thus xenin could hold real promise as a diabetes therapy. The biological actions of xenin, including its ability to augment insulin secretion, induce satiety effects, as well as restoring GIP sensitivity, earmark this peptide as an attractive antidiabetic candidate. This minireview will focus on the multiple biological actions of xenin, together with its proposed mechanism of action and potential benefits for the treatment of metabolic diseases such as diabetes.

## Introduction

Originally identified from human gastric duodenal and jejunal mucosal isolates,^1^ xenin, a naturally occurring 25-amino acid peptide, is synthesised from its 35-amino acid (aa) precursor pro-xenin.^2-4^ Interestingly, all 35-aa residues of yeast and mammalian alpha coat protein (COPA) are identical to that of pro-xenin.^3^ Biologically active xenin-25 (otherwise termed xenin) is then released following the action of pepsin on pro-xenin.^5,6^ Xenin has long been recognised as the human equivalent of the amphibian peptide xenopsin.^7^ Subsequent studies following on from original work by Feurle et al^1^ that evidenced xenin in human gastric mucosa, demonstrate that xenin can be further extracted from the gut of various other species including dog, rabbit, rat and pig.^6,8^ In keeping with the view that the gut harbours numerous important regulatory peptide hormones, the highest concentrations of xenin are found within the gastrointestinal system.^8^ In this regard, xenin is synthesised and secreted into the circulation from a subpopulation of chromogranin A-positive enteroendocrine K-cells,^9^ along with the incretin hormone, GIP, in response to food ingestion. However, Hamscher et al^8^ also identified xenin in other key organs in dogs, including hypothalamus, liver, kidney, heart, pancreas, testes and skin. More recent studies have also identified xenin immunoreactivity within the endocrine pancreas,^10^ suggesting local production and biological activity in this organ.

## Function, Potential Mechanism of Action and Therapeutic Application of Xenin

**Figure 1.**
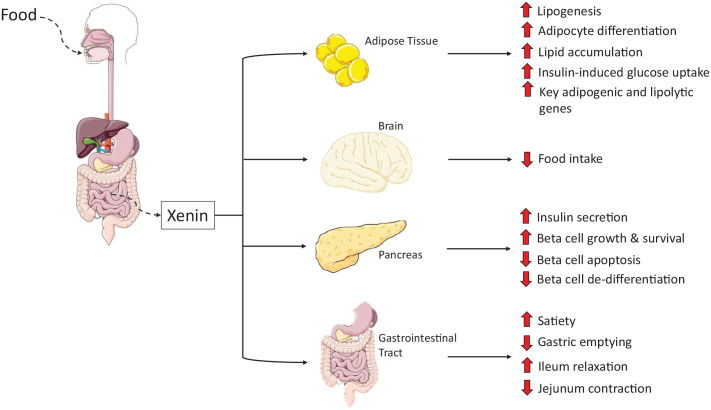
Representation of the main biological actions of xenin. The impact of xenin on adipose tissue, brain, pancreas and gastrointestinal tract are considered.

**Figure 2.**
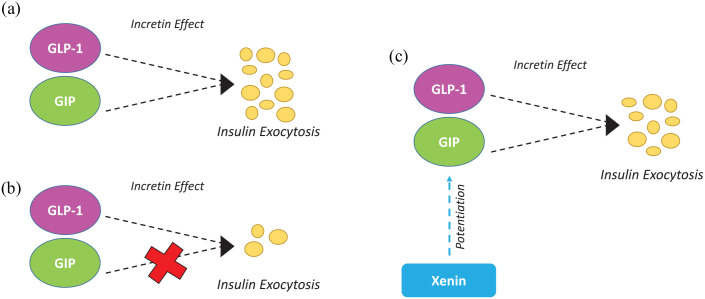
Representation of the incretin effect mediated by GLP-1 and GIP under normal and diabetic conditions, with perceived xenin benefits in diabetes. (a) The incretin response under normal physiology alongside (b) the perturbed incretin response in T2DM, with (c) xenin acting as a GIP potentiator to restore GIP sensitivity in T2DM.

Xenin possesses numerous important biological actions that have been established in various animal models, (see Figure 1; Table 1) which have previously been reviewed in depth.^4,6^ Briefly, key biological actions of xenin include control of energy balance and gastric transit,^1,6,11,12^ delay of gastric emptying in humans,^13^ appetite suppression,^6,13-16^ as well as regulating pancreatic exocrine and endocrine function.^1,4,6,9,16-22^ Xenin has also been shown to play a role in regulating normal bone physiology, potentially through indirect neural effects.^23^ Studies have also clearly revealed that xenin can potentiate the insulin-releasing capabilities of GIP (Figure 2), the incretin hormone co-secreted with xenin from intestinal K-cells,^19,21,24-26^ highlighting favourable attributes for the treatment of diabetes. Despite this established biological profile, a specific xenin receptor has yet to be identified. There is a suggestion that aspects of the biological actions of xenin may be mediated through activation of the neurotensin receptor, due to structural similarities between the 2 peptides.^27^ However, effects of xenin independent of neurotensin receptor activation have been demonstrated,^28^ highlighting the need for further detailed studies in this area. Finally, although there is no direct evidence for xenin induced benefits in type 1 diabetes mellitus, reduction of beta-cell apoptosis^10^ alongside positive actions on islet cell transdifferentiation,^29^ could be suggestive of positive effects of xenin in this disease state.

**Table 1.** Summary of evidence to support the main biological actions of xenin represented in Figure 1.

Species	Treatment	Main outcomes	References
Gastrointestinal actions			
Rodent	Experimental design • Dunken-hartley guinea pigs • Maximal efficacy of xenin-25 – 10^−6^ M	• In the jejunum ○ Small relaxation followed by a large contraction • In the colon ○ Myokinetic relaxation effect	Feurle et al^30^
Rodent	Experimental design • Xenin-25 (1 μM) at 15-20 min intervals	• Relaxation of rat ileum	Clemens et al^27^
Human	Experimental design • Constant intravenous infusions: 0-300 min • Infusion rates ○ Xenin @ 4 pmol/kg infusion ○ Xenin @ 12 pmol/kg – administered at the same relative flow rates as above	• Delay of gastric emptying in humans with and without T2DM • Reduction in postprandial glucose levels	Chowdhury et al^13^
Anorexigenic effects			
Chick	Experimental design Central effects on feeding: • Intracerebroventricular (ICV) injection of 0.75, 1.5 or 3.0 μg xenin. • Peripheral effects on feeding: • Intraperitoneal injection of avian saline, 0.2, 2.0 or 20.0 μg xenin dissolved in in 180 min fasted chicks Gastrointestinal transit rate: • Non-fasted chicks received the same ICV treatments as above. • Immediately after injection, chick was gavaged with feed slurry at a mass of 4.0% body weight	• Anorexigenic actions and delay gastrointestinal transit rate in chicks	Cline et al^11^
Rodent	Experimental design • Fasted (16 h) mice re-fed with pre-weighed food pellet for 1 h • Mice then given intraperitoneal injection of saline, xenin (50 μg/g bw) or urocortin (3 nmol/mouse) • Rate of gastric emptying was calculated as follows: Gastric emptying (%) = {1 − (wet weight of food recovered from the stomach/wet weight of food intake)} × 100^12^ • The effect of xenin on food intake was examined in ad libitum–fed wild-type mice. Mice were injected intraperitoneally with xenin (50 μg/g bw) or saline and cumulative food intake was measured 1, 2, 4, 6, 8, 12, 18 and 24 h after injection^15^ • Mice were fasted for 12 h before subcutaneous injection of 50, 100 or 500 nmol/kg xenin. Mice were then allowed free access to normal chow. Cumulative food intake was measured at 30, 60, 60 and 120 min post injection^18^	• Reduction of gastric emptying by 93% and induction of satiety	Kim and Mizuno,^12^ Alexiou et al,^14^ Leckstrom et al,^15^ Taylor et al,^18^ Cooke et al,^31^ and Bhavya et al^32^
Adipose Tissue			
Rodent	Experimental design • *Ad libitum* fed mice received 2 ICV injections of xenin (5 μg) at 10:00 h and 22:00 h • Body weight and food weight were measured immediately prior to the first injection and 24 h after the first injection. Mice were euthanised 12 to 14 h after the second injection Epididymal adipose tissues and skeletal muscles were collected for RNA and protein analyses	• Increased expression of lipolytic markers	Bhavya et al^32^
3T3-L1 mouse adipocyte cell line	Experimental design • Immortalised 3T3-L1 fibroblasts differentiated 2 days post confluence in the absence or presence of xenin-25-Gln (10^−6^ M). Test peptides were added only during the key growth phase when the differentiation cocktail was present • Glycerol release, glucose uptake and gene expression were assessed	• Increased glycerol release • Key adipogenic and lipolytic genes upregulated • Stimulated insulin-induced glucose uptake	English et al^33^

### GIP potentiation

Resistance to the biological actions of GIP is a hallmark of type 2 diabetes mellitus, with the GIP-mediated incretin effect being severely diminished in people with diabetes (Figure 2).^34^ However, despite the well-known importance of the incretin effect to regulate normal blood glucose levels,^35^ established treatments for type 2 diabetes fail to address this issue. Indeed, incretin-based therapeutics focus largely on augmenting the biological actions of the sister incretin glucagon-like peptide-1 (GLP-1). However, recent exciting clinical findings with a dual-acting GLP-1 and GIP receptor hybrid peptide exhibiting strong bias towards the GIP receptor,^36^ suggests that GIP resistance in type 2 diabetes is surmountable. In this regard, xenin has been shown to potentiate the insulinotropic actions of GIP in rodent models of diabetes.^18-22,24-26^ Whilst the precise mechanism of xenin-induced GIP potentiation remains to be fully elucidated,^25,27,37^ it may be linked to acetylcholine M3 receptor signalling on pancreatic beta cells.^25^ However, there is also good evidence for a direct effect of xenin on beta cells,^6^ that is reinforced by knowledge that xenin is produced and secreted locally within islets.^10^

### Appetite suppression

Several studies have demonstrated the role of xenin in regulating energy intake. Administration of xenin reduces calorie consumption and delays gastric emptying in mice, rats, chicks and humans^6,11,13-15,18,31^ suggesting xenin may act directly on the gastrointestinal tract to induce satiety. This effect may occur through receptor binding at nerve terminal ends, which then influences the nucleus of the solitary tract anorexigenic activity, or hypothalamic receptors involved in energy homeostasis.^38^ Indeed, hypothalamic neurons appear to have direct involvement in regulation of calorie intake following intraperitoneal administration of xenin, suggesting centrally mediated effects.^15^ Interestingly, more recent studies have characterised xenin activity in both peripheral and central regions linked to regulating feeding in goldfish, to induce anorexigenic actions.^39^ It has also been demonstrated that xenin, when administered intracerebroventricularly in rats or peripherally in mice, may act through CRH-dependent signalling pathways to regulate food intake.^38^ However, it has been established that anorexic effects of xenin are independent of both the leptin- and melanocortin-dependent signalling pathways.^15^

### Lipid metabolism

In addition to its role in reducing food intake, xenin has also been shown to cause alterations in the expression of genes involved in lipid metabolism, as well as proteins found within white adipose tissue.^32^ There was an original hypothesis that xenin acts on adipose tissue to stimulate lipolysis, and that xenin may hold promise as an anti-obesity therapy by reducing adipose fat depots, but such observations were somewhat inconsistent.^32^ Thus, English et al^33^ recently revealed direct lipogenic and lipolytic actions of xenin in 3T3-L1 adipocytes, whilst also promoting adipocyte differentiation in 3T3-L1 pre-adipocytes, through alterations in gene expression of LPL and FASN, key promoters of 3T3-L1 differentiation.^33^ The effects of xenin to positively modulate lipolysis, lipogenesis and adipocyte differentiation are likely modulated through NTRS1 activation on the AKT/PI3K pathway.^33^ However, it should be noted that the actions of xenin on lipid metabolism are still not well defined and require more detailed study, especially in light of some conflicting observations.^32,33^

### Pancreas

Immunoreactivity of xenin has been identified in human pancreatic extracts,^8^ where concentrations increased following pepsin digestion.^8^ More recently, immunohistochemical-based methods demonstrated expression of xenin in both alpha- and beta cells, with both arginine and glucose acting as a stimulus for xenin secretion from the islet.^10^ Numerous biological roles of xenin in the pancreas have already been recognised, including secretion of insulin and glucagon, as well as effects of secretory activity in the exocrine pancreas.^6^ In addition, xenin exerts beneficial effects on beta cell growth and protection against apoptosis,^6^ with obvious therapeutic benefit in the context of diabetes. Moreover, recent studies in insulin-deficient *Ins1*^Cre/+^; *Rosa26-eYFP* transgenic mice with islet cell lineage tracing capabilities reveal positive effects of xenin on islet cell differentiation, including maintenance of beta cell identity and prevention of beta cell de-differentiation.^29^ These positive effects on islet cell architecture may be related to potentiation of the biological actions of GIP, since GIP has established benefits on beta cell growth and survival, as well as transdifferentiation.^40-43^ The mechanisms related to these xenin-mediated pancreatic islet actions are somewhat disputed however, with proposed importance of both direct and indirect actions. Thus, xenin has been shown to directly stimulate glucagon and insulin secretion in vitro when applied to cultured pancreatic alpha- and beta-cells, respectively.^17^ These direct receptor-mediated actions are strengthened by evidence of local xenin production and secretion within pancreatic islets.^10^ On the other hand, there are also reports to suggest that xenin does not directly enhance GIP-mediated insulin exocytosis, with these effects stimulated through activation of acetylcholine containing enteric neurons that are in direct contact with the pancreas.^25^

### Polycystic ovary syndrome

Insulin resistance is an established pathological feature of type 2 diabetes mellitus, with polycystic ovary syndrome (PCOS) also closely associated with obesity and insulin resistance.^44^ Thus, similar to diabetes, previous research has defined a relationship between xenopsin-related-peptide-1 and PCOS, where the levels of xenopsin-related-peptide-1 were significantly elevated in PCOS patients when compared to controls.^45^ In this regard, serum xenin concentrations are significantly elevated in women with PCOS compared to women with no menstrual cycle abnormalities.^46^ However, as with diabetes,^6^ the precise impact of xenin in PCOS and its pathophysiology remains to be fully elucidated. When viewed together, the above diverse biological actions of xenin emphasise potential for targeting related pathways for the amelioration of insulin resistance and related disease such as diabetes and PCOS.

## Truncated Xenin Peptides and Analogues

Naturally occurring peptides such as xenin have many therapeutic advantages over small molecules, including their diversity, safety, ease of synthesis, along with minimal risk of drug-drug interactions.^47^ Naturally occurring peptides also have a high binding affinity towards a broad, but specific range of therapeutic targets and are often very potent, resulting in enhanced efficacy, selectivity and specificity, even at lower therapeutic doses.^48^ Therefore, peptide therapeutics are of great interest for drug developers. However, the clinical use of peptides is hindered by certain disadvantages, including their instability and susceptibility to enzymatic degradation, reduced oral bioavailability, limited cell membrane permeation and rapid renal clearance.^49^ Fortunately, these limitations can be largely overcome through structural modification of the peptide,^49-53^ which has been demonstrated for xenin, as discussed below.

Stable analogues of xenin (Tables 2 and 3) with preserved or even enhanced bioactivity have been developed.^6^ Many of these xenin analogues possess notable beneficial metabolic effects in pre-clinical models of diabetes-obesity,^16,22,54^ which has been reviewed in detail previously.^6^ However, the use of truncated peptide fragments of xenin that retain the full biological actions of the parent peptide, could enhance therapeutic promise by making peptide synthesis easier and cheaper, as well as facilitating possible non-injectable peptide drug delivery.^55,56^ An earlier comprehensive exploration identified the degradation profile of xenin in mouse plasma, revealing the following C-terminally truncated metabolites; xenin 9-25, xenin 11-25, xenin 14-25 and xenin 18-25 (where xenin 18-25 represents xenin-8).^20^ Subsequent characterisation revealed that only xenin-8 possessed biological activity equivalent to the parent peptide.^20^ Indeed, this truncated octapeptide has long been recognised as a naturally occurring and biologically active derivative of xenin,^17,57,58^ that retains full insulinotropic actions.^20^ Furthermore, amino acid substitution of the Lys and Arg residues for Gln in xenin-8, resulted in production of a fully enzymatically stable octapeptide that retained full gluco-regulatory and antidiabetic actions as full-length xenin.^16^ Subsequent recent research has now confirmed bioactivity of xenin-6 (xenin 20-25) at the level of the endocrine pancreas.^26,50^ Moreover, modification of xenin-6 through introduction of a reduced pseudopeptide bond between amino acid residues Lys-20 and Arg-21, to create xenin-6-psi, further increased bioactivity of this truncated peptide.^26,50^ Intriguingly, xenin-6-psi exerted potent metabolic actions in diabetic rodents and prominently augmented the biological actions of the incretin hormone GIP.^26^ Thus, it appears that the 6 C-terminal residues of xenin are sufficient to facilitate receptor binding and activation of the full repertoire of xenin cell signalling pathways.

**Table 2.** Amino acid sequences of xenin-25 as well as its related stable analogues and naturally occurring fragment peptides.

Peptide	Amino acid sequence	References
Xenin-25	M-L-T-K-F-E-T-K-S-A-R-V-K-G-L-S-F-H-P-K-R-P-W-I-L-OH	Feurle et al^1^
Xenin-25-Gln	M-L-T-Q-F-E-T-Q-S-A-Q-V-Q-G-L-S-F-H-P-Q-Q-P-W-I-L-OH	Parthsarathy et al^22^
Xenin-25[Lys^13^PAL]	M-L-T-K-F-E-T-K-S-A-R-V-K-(N-ε-(γ-GLU(hexadecanoyl))-G-L-S-F-H-P-K-R-P-W-I-L-OH	Gault et al^21^
Xenin 9-25 (Xenin-17)	S-A-R-V-K-G-L-S-F-H-P-K-R-P-W-I-L-OH	Martin et al^20^
Xenin 11-25 (Xenin-15)	R-V-K-G-L-S-F-H-P-K-R-P-W-I-L-OH	Martin et al^20^
Xenin 14-25 (Xenin-12)	G-L-S-F-H-P-K-R-P-W-I-L-OH	Martin et al^20^
Xenin 18-25 (Xenin-8)	H-P-K-R-P-W-I-L-OH	Martin et al^20^
Xenin 18-25-Gln	H-P-Q-Q-P-W-I-L-OH	Martin et al^16^
Xenin 20-25 (Xenin-6)	K-R-P-W-I-L-OH	Craig et al^26^ and Feurle et al^50^
Xenin-6-psi	K-(CH_2_NH)-R-P-W-I-L-OH	Craig et al^26^ and Feurle et al^50^

**Table 3.** Summary of study design and main experimental outcomes from studies with fragment peptides of xenin-25.

Species	Treatment	Main outcomes	References
In vitro and rodent	Experimental design • For food intake studies, fasted (18 h) mice were given intraperitoneal (i.p) injections of either saline vehicle (0.9% w/v NaCl), xenin-8 or xenin-8-Gln at a dose of 500 nmol/kg bw. Cumulative food intake measured over 120 min • For glucose homeostasis and insulin secretory studies, blood glucose and plasma insulin concentrations were measured immediately prior to and 15, 30 and 60 min after i.p. administration of glucose alone (18 mmol/kg bw) or in combination with either xenin 18 to 25 or xenin 18-25 Gln (each at 25 nmol/kg bw) in non-fasted mice	• Concentration-dependently stimulated insulin secretion • Enhanced glucose-induced insulin release	Martin et al^16^
Rodent	Experimental design • Twice daily i.p. injections of saline vehicle, xenin-8 or xenin-8-Gln (both at 25 nmol/kg bw) for 21 days in HFF mice • Energy intake, body weight, non-fasting blood glucose and plasma insulin concentrations were assessed during the 21 days • At the end of the treatment period, i.p. glucose tolerance (18 mmol/kg bw), biological response to GIP (18 mmol/kg glucose in combination with native GIP (25 nmol/kg); i.p.) and insulin sensitivity (15 U/kg bw; i.p.) tests were performed	• Both treatment regimens ○ Elevated circulating plasma insulin concentrations ○ Improved insulin sensitivity • Xenin-8-Gln ○ Improved glucose tolerance ○ Augmented GIP-mediated glucose-lowering and insulin-releasing effects	Martin et al^16^
Rodent	Experimental design • For food intake studies, fasted (18 h) lean mice were given intraperitoneal (i.p) injections of either saline vehicle (0.9% w/v NaCl) or Ψ-xenin-6 at a dose of 25 or 250 nmol/kg bw. Cumulative food intake was measured at 30 min intervals for 180 min • For acute effects of peptides on glucose tolerance and insulin secretion, blood glucose and plasma insulin concentrations were determined immediately prior to and 15, 30, 60 and 105 min after i.p. injection of glucose alone (18 mmol/kg bw) or in combination with test peptides (25 nmol/kg bw), as well as test peptides together with GIP (25 nmol/kg bw) in 4 h fasted mice • To assess duration of peptide action, mice were administered saline vehicle or test peptides (25 nmol/kg bw) at 2, 4, 8 or 12 h prior to an i.p. glucose challenge (18 mmol/kg bw) and blood glucose measured	• Significantly reduced glucose levels • Enhanced glucose-induced insulin release • Enhanced the glucose-lowering action of GIP • Exhibited satiety actions	Craig et al^26^
Rodent	Experimental design • Oral sitagliptin phosphate monohydrate once daily (50 mg/kg bw), intraperitoneal (i.p.) Ψ-xenin-6 twice daily (25 nmol/kg bw) or a combination of both compounds for 18 days in HFF mice • Energy intake, body weight, non-fasting blood glucose and plasma insulin concentrations were assessed at regular intervals • At the end of the treatment period, i.p. glucose tolerance (18 mmol/kg bw; 18 h-fasted mice), insulin sensitivity (25 U/kg bovine insulin; i.p.; non-fasted mice) and pyruvate tolerance (2 g/kg sodium pyruvate; i.p.; 18 h-fasted mice) tests were performed • HOMA-IR, fasting glucose (mmol/L) × fasting insulin (mU/L)/22.5, was also calculated as a surrogate marker of insulin resistance • Terminal analyses included extraction of pancreatic tissue for determination of pancreatic insulin content. In addition, liver tissue was processed for hepatic gene expression by qPCR after total RNA extraction	Ψ-xenin-6 alone • Reduced weight gain • Reduced glucose levels as well as improved glucose tolerance and insulin sensitivity. • Positive effects on pancreatic islet architecture Ψ-xenin-6 and sitagliptin: • Prominent benefits on circulating glucose and insulin levels • Improvements in attenuating gluconeogenesis • Benefits on pancreatic islet architecture • Improved insulin sensitivity	Craig et al^59^

## Dual and Triple Acting Therapeutic Approaches That Incorporate Xenin Elements

As noted above, truncated xenin peptides retain bioactivity and have promising antidiabetic actions.^20,26,50^ However, in such a multi-factorial disease as type 2 diabetes mellitus, monotherapy does not appear to adequately control glycaemia over the longer-term. Thus, multi-targeting unimolecular hybrid peptides, designed to simultaneously modulate multiple signalling pathways are now thought to offer superior therapeutic efficacy than single targeted compounds.^60^ Indeed, data emerging from recent clinical studies with a dual-acting GLP-1/GIP compound, Tirzepatide (LY3298176), developed by Lily, with strong bias towards the GIP receptor, fully support this notion.^61^ Data from phase 1 and 2 studies were extremely promising, with the compound now entering SURPASS phase 3 clinical trials to determine long-term efficacy and safety.^62^ Initial proof-of-concept for utilisation of multi-acting hybrid peptides comes from the naturally occurring dual agonist oxyntomodulin (OXM), that activates both GLP-1 and glucagon receptor pathways.^63^ More recent studies demonstrate the opportunity of linking together individual bioactive peptide domains of different peptides, or engineering unique amino acid sequences that incorporate binding capabilities of 2 or more regulatory peptides, to create multi-targeting hybrid peptides.^64-66^

With regards to type 2 diabetes mellitus, Gault et al^67^ initially indicated that a GLP-1 and GIP preparation, that combined long-acting acylated version of the parent peptides, displayed enhanced glucose-lowering and insulinotropic actions in animal models of diabetes. This being despite earlier observations that combined administration of individual enzymatically stable, but non-acylated GIP and GLP-l mimetics was not associated with benefits beyond that of either peptide alone,^68-70^ however this could be related to differences in treatment regimens or animal models employed. Following on from this, a triple acting hybrid peptide comprising GLP-1, GIP and glucagon was developed that offered some improvements in preclinical models of obesity-diabetes when compared to parent peptides.^71^ In addition, 2 separate CCK/GLP-1 fusion peptides have been characterised revealing notable benefits on appetite suppression, insulinotropic effects as well as beta cell function and morphology.^64,72^ Furthermore, numerous other dual- and triple-acting hybrid peptides have been developed that clearly advocate the therapeutic benefits of single peptide-based drugs capable of positivity modulating more than 1 receptor pathway for the treatment of diabetes.^65,66,73-75^ Tschöp et al^75^ demonstrated that novel unimolecular combination therapies have superior efficacy, compared to current therapeutic options, thus having potential to reverse obesity and type 2 diabetes.

In terms of incorporating xenin into multi-acting hybrid peptides (Tables 4 and 5), this was first demonstrated in 2017 through a GIP/xenin entity, namely (DAla^2^)GIP/xenin-8-Gln.^54^ Subsequent work with (DAla^2^)GIP/xenin-8-Gln has highlighted that twice-daily administration in high fat fed mice for 28 days significantly reduced food intake and body weight, with associated reductions in circulating glucose concentrations and HbA_1c_ levels, whilst improving glucose tolerance and insulin sensitivity.^76^ Similar, but somewhat less striking antidiabetic effects were noted in *db/db* mice given (DAla^2^)GIP/xenin-8-Gln, demonstrating that the positive antidiabetic actions are transferable across diverse aetiologies of type 2 diabetes mellitus.^76^ Remarkably, the same study also demonstrated long-acting positive metabolic effects of (DAla^2^)GIP/xenin-8-Gln following 14-day cessation of treatment.^76^ This could suggest positive metabolic reprogramming induced by co-activation of GIP and xenin receptor pathways in keeping with positive effects on beta cell function and integrity,^6,29,43^ and represents a potential benefit for future antidiabetic therapy. Such observations are extremely important moving towards the clinical setting given the complex aetiology and progressive nature of type 2 diabetes mellitus in humans.^77^ Subsequent investigations characterised a novel GLP-1/xenin hybrid peptide (exendin-4/xenin-8-Gln) that exhibited positive antidiabetic actions in high fat fed mice,^78^ highlighting positive effects of combined modulation of GLP-1 and xenin related signalling pathways in diabetes. Hasib et al^78^ also demonstrated the potential of combined modulation of GLP-1, gastrin and xenin signalling pathways,^78,79^ which was superior to the previously described dual-acting fusion peptide incorporating GLP-1 and gastrin only, namely ZP3022.^80^

**Table 4.** Amino acid sequences of xenin incorporated multi-acting hybrid peptides.

Peptide	Amino acid sequence	References
(DAla^2^)GIP/xenin-8-Gln	Y-[DA]-E-G-T-F-I-S-D-Y-S-I-A-M-H-P-Q-Q-P-W-I-L-OH	Hasib et al^54^ and Pathak et al^74^
Exendin-4/xenin-8-Gln	H-G-E-G-T-F-T-S-D-L-S-K-Q-M-E-E-E-A-V-R-L-F-I-E-W-L-K-N- AEEAc – AEEAc-H-P-Q-Q-P-W-I-L-OH	Craig et al^76^
Exendin-4/gastrin/xenin-8-Gln	H-G-E-G-T-F-T-S-D-L-S-K-Q-M-E-E-E-A-V-R-L-F-I-E-W-L-K-N- AEEAc – AEEAc-Y-G-W-L-D-F- AEEAc – AEEAc-H-P-Q-Q-P-W-I-L-OH	Hasib et al^81^
Exendin-4(Lys^27^PAL)/gastrin/xenin-8-Gln	H-G-E-G-T-F-T-S-D-L-S-K-Q-M-E-E-E-A-V-R-L-F-I-E-W-L-K(γ-Glu-palm)-N-AEEAc-AEEAc-Y-G-W-L-D-F-AEEAc-AEEAc-H-P-Q-Q-P-W-I-L-OH	Hasib et al^78^

**Table 5.** Summary of study design and main experimental outcomes from studies with xenin incorporated multi-acting hybrid peptides.

Species	Treatment	Main outcomes	References
Rodent	Experimental design • Twice daily i.p. injections of saline vehicle, (DAla^2^)GIP/xenin-8-Gln (25 nmol/kg bw), exendin-4 (25 nmol/kg bw), or a combination of both peptides for 28 days in HFF mice, followed by 14 days cessation of treatment • Energy intake, body weight, non-fasting blood glucose and plasma insulin concentrations were assessed at regular intervals • At the end of the treatment period, i.p. glucose tolerance (18 mmol/kg bw; 18 h-fasted mice) and insulin sensitivity (25 U/kg bovine insulin; i.p.; non-fasted mice) tests were performed. Metabolic responses to acute re-administration of respective treatment regimens together with glucose was also examined • On day 28 observations were continued in a sub-group (n = 6) of mice following cessation of treatment regimens for a further 14 days, with assessment of the same parameters as documented above	(DAla^2^)GIP/xenin-8-Gln • Reduction in food intake, body weight, circulating glucose and HbA1_C_ • Improved glucose tolerance and insulin sensitivity • Improved pancreatic architecture	Craig et al^76^
Rodent	Experimental design • Twice daily i.p. injections of saline vehicle, (DAla^2^)GIP/xenin-8-Gln (25 nmol/kg bw), exendin-4 (25 nmol/kg bw), or a combination of both peptides for 28 days in *db/db* mice • Energy intake, body weight, non-fasting blood glucose and plasma insulin concentrations were assessed at regular intervals • At the end of the treatment period, i.p. glucose tolerance (18 mmol/kg bw; 18 h-fasted mice) and insulin sensitivity (50 U/kg bovine insulin; i.p.; non-fasted mice) tests were performed	• (DAla^2^)GIP/xenin-8-Gln in combination with exendin-4 was required to induce beneficial effects on glucose tolerance, insulin sensitivity	Craig et al^76^
Rodent	Experimental design: • Twice daily i.p. injections of saline vehicle (0.9% w/v NaCl), exendin-4, exendin-4/gastrin/xenin-8-Gln alone and in combination with (DAla2)GIP (each peptide at 25 nmol/kg bw) for 21 days in HFF mice • Cumulative food intake, body weight, non-fasting glucose and insulin concentrations were monitored at regular intervals • Circulating glucagon, amylase activity and blood lipid profile were assessed at the end of the treatment period • Glucose tolerance (18 mmol/kg bw; i.p.), metabolic response to GIP (18 mmol/kg glucose in combination with native GIP (25 nmol/kg); i.p.) and insulin sensitivity (25 U/kg bw; i.p.) tests were performed at the end of the treatment period • On day 21 locomotor activity and energy expenditure were assessed	• Reduced circulating glucose and increased plasma insulin concentrations • Improved glucose tolerance, insulin sensitivity and metabolic response to GIP • Reduced LDL-cholesterol and body fat mass • Normalised pancreatic islet and beta-cell area • Increase in energy expenditure and locomotor activity in mice treated with exendin-4/gastrin/xenin-8-Gln in combination with (DAla^2^)GIP	Hasib et al^81^
Rodent	Experimental design • Dosing Regimen: Twice-daily injections of saline vehicle, exendin-4/gastrin, exendin-4/gastrin/xenin-8-Gln, or exendin-4(Lys^27^PAL)/gastrin/xenin-8-Gln (each at 25 nmol/kg bw; ip) for 31 days in *ob/ob* mice • Energy intake, body weight, non-fasting blood glucose and plasma insulin concentrations were assessed at regular intervals • Plasma glucagon, amylase activity, 24-h glucose profile and whole blood HbA_1c_ were measured on day 31 • At the end of the treatment period, glucose tolerance (18 mmol/kg bw; ip), metabolic response to GIP (18 mmol/kg glucose in combination with native GIP [25 nmol/kg]; ip) and insulin sensitivity (50 U/kg bw; ip) tests were conducted • Percentage body fat and pancreatic insulin content were also determined	• Decreased food intake, glucose and HbA_1c_ concentrations • Enhanced circulating and pancreatic insulin levels • Improved glucose tolerance and glucose-induced insulin secretion • Enhanced metabolic response to GIP and the glucose-lowering actions of insulin	Hasib et al^79^

More recent work has explored the possibility of Ψ-xenin-6 to enhance the antidiabetic efficacy of the established dipeptidyl peptidase-4 (DPP-4) inhibitor drug sitagliptin.^59^ Multiple metabolic advantages of combined Ψ-xenin-6 and sitagliptin therapy were observed, including benefits on body weight, circulating glucose and insulin along with additional enhancements to reduce gluconeogenesis and improve pancreatic islet architecture.^59^ Additional related studies have demonstrated how specifically elevating xenin concentrations through use of the methionine aminopeptidase inhibitor 2, TNP-470, can also augment the antidiabetic efficacy of sitagliptin.^82^ Moreover, as well as increasing xenin secretion, TNP-470 is a putative anti-obesity agent,^83-85^ highlighting obvious benefits of this treatment modality in obesity-driven forms of diabetes. Given xenin has confirmed GIP-potentiating actions, the combination of therapies that increase xenin bioactivity alongside established DPP-4 inhibitor drugs clearly warrants further consideration as a novel therapeutic option in the management of type 2 diabetes mellitus in humans.

## Concluding Remarks

This minireview highlights the diverse biological actions of xenin, as well as the therapeutic potential for xenin and related truncated metabolites for diabetes and related disorders. Future studies are required to fully understand the signalling pathways and mechanisms involved in the insulinotropic, GIP-potentiating and anorexigenic actions of xenin, as well as the role of xenin signalling within benefits of associated hybrid peptides. Clarification of whether or not a specific xenin receptor exists is key in this paradigm. Nevertheless, xenin possesses a promising therapeutic repertoire that may result in the development of a safe, effective, long-acting and cost-effective therapy for obesity-diabetes.

Due to the multifactorial nature of type 2 diabetes mellitus, monotherapy is often not an effective treatment option. Thus, combination therapy or hybrid peptides have the potential to emerge as leading therapeutic approaches for this disease. Both approaches show promise with xenin-based therapies, demonstrating obvious advantages over monotherapy that is highly favourable moving towards the clinic.^54,76,78,79^ However, future studies are required to fully understand the mechanisms and pathways associated with satiety effects, insulinotropic and GIP-potentiating actions to gain a better understanding of the role of xenin and overall therapeutic potential of these hybrid peptides. Further to this, recent studies have highlighted the stability and metabolic benefits of Ψ-xenin-6 alone,^26^ and in combination with established anti-diabetic therapies.^59^ To date, hybrid peptides that contain a xenin element have focussed on xenin-8 sequences, but utilisation of xenin-6 peptides, particularly xenin-6-psi, could offer distinct advantages over this approach. In terms of potential side effects of xenin-based therapeutics, the only notable reported side effect following xenin infusion in humans was mild diarrhoea.^13^ There is also lack of any obvious side effects in rodents following sustained xenin administration in numerous studies.^54,76^ Thus, xenin appears to have side-effect profile similar to that of established GLP-1 therapeutics, namely mild gastrointestinal adverse events, with GLP-1 mimetics now well-established as important anti- obesity and -diabetes drugs in man.^86,87^ However, further dose-response studies are still required in human volunteers to uncover the complete adverse side effect profile of xenin. Ultimately, xenin-based therapies need to be further assessed in the human setting to confirm translatability of the many positive findings from preclinical trials,^21,26,59,76,78,79,82,83^ and progress benefits towards the clinic.
